# Clinical Profile of Patients With Isolated Lateral Rectus Palsy in Adults

**DOI:** 10.7759/cureus.105158

**Published:** 2026-03-13

**Authors:** Ramya Karjol, Renuka Kattimani, Sachin Tammannavar

**Affiliations:** 1 Department of Ophthalmology, Shri B. M. Patil Medical College Hospital and Research Centre, Vijayapura, IND; 2 Department of Ophthalmology, Employees’ State Insurance Corporation (ESIC) Medical College and Hospital, Kalaburgi, IND; 3 Department of Ophthalmology, Karwar Institute of Medical Sciences, Karwar, IND

**Keywords:** abducens nerve palsy, cranial nerve vi, horizontal diplopia, isolated lateral rectus palsy, microvascular ischemia

## Abstract

Background

Isolated lateral rectus (LR) palsy is a common cranial nerve palsy with a variety of causes. Horizontal diplopia is frequently caused by isolated LR palsy, a common cranial nerve palsy in adults. The abducens nerve is vulnerable to a variety of vascular, inflammatory, traumatic, viral, and compressive diseases because of its lengthy intracranial journey. This study aims to describe the clinical profile, etiological distribution, and outcomes in adults with isolated LR palsy.

Methodology

This retrospective observational study included 29 consecutive adults diagnosed with isolated LR palsy. Demographic characteristics, clinical presentation, investigation findings, etiological diagnoses, and recovery outcomes during follow-up were systematically recorded and analyzed using descriptive statistical methods.

Results

The mean age was 48.17 years (range = 19-85 years), with a male-to-female ratio of 15:14. Etiologies included diabetes-related microvascular ischemia in seven (24.1%) patients, hypertension-related microvascular ischemia in seven (24.1%) patients, infection with inflammation in four (13.8%) patients, trauma in four (13.8%) patients, idiopathic causes in two (6.9%) patients, tumor in one (3.4%) patient, cavernous sinus thrombosis in three (10.3%) patients, and microvascular ischemia associated with both diabetes mellitus and hypertension in one (3.4%) patient. At the last follow-up, 19 (65.5%) patients had complete recovery, two (6.8%) had partial recovery, one (3.4%) had no recovery, and seven (24.1%) were lost to follow-up.

Conclusions

Microvascular ischemia associated with diabetes mellitus and hypertension was the leading cause of isolated LR palsy in adults. Most patients demonstrated improvement during follow-up, whereas traumatic and compressive etiologies showed relatively poorer outcomes. Careful systemic evaluation and targeted neuroimaging are important in patients with atypical presentations or suspected non-microvascular causes. These findings emphasize the importance of systematic clinical assessment, etiological evaluation, and follow-up in adults presenting with isolated LR palsy.

## Introduction

Isolated lateral rectus (LR) palsy, secondary to the dysfunction of the sixth cranial nerve, also known as the abducens nerve, is a very common neuro-ophthalmic clinical condition and a very common cause of horizontal diplopia in the adult population [1]. The LR muscle is responsible for the abduction of the globe, and dysfunction of the LR muscle causes characteristic clinical features: esotropia in the primary position and limitation of the outward gaze on the side with the affected muscle, resulting in binocular horizontal diplopia with worsening during the gaze directed to the affected eye [2]. Because the abducens nerve has the longest intracranial course of all the ocular motor nerves and passes from the pontine nucleus through the subarachnoid space, cavernous sinus, and superior orbital fissure before entering the orbit, the abducens nerve is especially vulnerable to a wide range of pathological insults [3].

In adults, isolated LR palsy can have different etiologies that reflect both systemic and local factors impacting the nerve along its anatomical pathway [4]. Microvascular ischemia associated with diabetes mellitus and hypertension is a well-recognized and common cause of isolated LR palsy, particularly in older adults with established vascular risk factors [5]. Traumatic injury, inflammatory disorders, infectious processes, intracranial or orbital neoplasms, and conditions associated with increased intracranial pressure are also important causes, and they may compromise the sixth cranial nerve at different points along its anatomical course [6]. Despite improvements in diagnostic evaluation, there is still a subset of patients for whom no definite cause can be identified despite thorough clinical evaluation and appropriate investigations, and these cases are classified as idiopathic isolated LR palsy [7].

The clinical picture of isolated LR palsy depends on the cause, the age of the patient, and the accompanying systemic diseases [8]. Some patients have a sudden onset of diplopia with conspicuous ocular deviation, while some patients may have a gradual onset or progressive symptoms. The presence of systemic risk factors, including diabetes mellitus and hypertension, strongly implies the existence of a microvascular ischemic mechanism, while associated neurologic signs or pain and/or progressive worsening may suggest compressive, inflammatory, or infectious causes [9]. The pattern of natural history and recovery is also dependent on the cause, with microvascular palsies sometimes showing spontaneous improvement over weeks to months, while in traumatic, neoplastic, or inflammatory etiologies, it may be delayed, incomplete, or absent [10].

A detailed clinical profile of patients with isolated LR palsy is therefore necessary for early diagnosis, appropriate investigations, and early management [3]. Careful assessment of demographic features, clinical features, ocular examination, and systemic risk factors assists clinicians in narrowing down the differential diagnoses and the priority investigation [1]. As the etiological spectrum is important to understand benign, self-limiting conditions from possibly serious or life-threatening disorders such as intracranial tumors, cavernous sinus pathology, or severe infections requiring urgent intervention [6]. Accurate identification of the cause enables it to be appropriately managed and referred to, where necessary [2].

Comprehensive assessment of patients with isolated LR palsy usually consists of detailed medical history, careful ocular and neurological examination, and selective use of laboratory investigations and neuroimaging depending upon the clinical suspicion [5]. Knowledge of anticipated outcomes according to etiology is of value to the clinician in advising the patient of prognosis and planning follow-up strategies [9]. Additionally, understanding of normal recovery timeframes helps identify unusual cases that do not follow the expected normal volume of recovery and could merit further evaluation and/or alternative management approaches [7].

Despite the clinical relevance of isolated LR palsy, there is considerable heterogeneity in reported etiological distributions and outcomes in different studies, reflecting differences in study populations, diagnostic criteria, and healthcare settings [8]. Population-based and hospital-based studies have provided valuable knowledge, but population data from many parts of the world are still lacking [10]. Systematic assessment of clinical profiles in defined adult populations can thus contribute to a better understanding of prevailing causes and patterns of recovery and to evidence-based clinical decision-making [4].

Isolated LR palsy in adults is an important clinical entity that requires systematic evaluation to ensure correct diagnosis and appropriate treatment [1]. The etiological spectrum, clinical manifestations, and patterns of recovery are important to understand to guide the diagnosis, risk stratification, and follow-up management [6]. Careful documentation and analysis of clinical features of affected patients can aid in the refinement of existing diagnostic approaches, support the identification of serious underlying conditions in a timely manner, and inform the optimal management pathway in routine clinical practice [3].

Objectives of the study

This study aims to analyze the clinical profile of isolated LR palsy in adults, focusing on etiological factors, clinical presentation, and recovery outcomes during follow-up. The primary outcome of the study was recovery of LR function and resolution of diplopia, while secondary outcomes included the etiological distribution, demographic characteristics, and clinical presentation of patients with isolated LR palsy. By examining these aspects in an organized way, the study aims to contribute to the existing literature and to improve the clinical assessment, investigation, and management of adults who present with isolated LR palsy.

## Materials and methods

Study design

This study was conducted as a retrospective observational study to systematically assess adults presenting with isolated LR palsy. Medical records of patients diagnosed with isolated LR palsy during the study period were reviewed to obtain information on demographic characteristics, clinical presentation, etiological factors, investigations, management, and follow-up outcomes. This design enabled systematic evaluation of the clinical profile, underlying etiological factors, and recovery outcomes in patients with isolated LR palsy.

Study setting and duration

The study was conducted at the Department of Ophthalmology of a tertiary care hospital in North Karnataka. The hospital functions as a referral center for surrounding districts and provides ophthalmic care to a diverse patient population. Patient records from January 2025 to December 2025 were reviewed, and all eligible cases presenting within this time frame were included in the study.

Study population

The study included 29 adult patients diagnosed with isolated LR palsy. Adults were defined as individuals older than 18 years of age. Consecutive sampling was used, ensuring that all eligible patients presenting during the study period were included to minimize selection bias.

Inclusion and exclusion criteria

Patients older than 18 years who were diagnosed with isolated LR palsy were eligible for inclusion. Isolated palsy was defined as sixth cranial nerve involvement without clinical evidence of dysfunction of other cranial nerves.

Patients were excluded if they had multiple cranial nerve palsies, a history of previous strabismus surgery, or congenital ocular motor palsies, as these conditions could introduce heterogeneity and act as confounding factors influencing clinical presentation or recovery outcomes.

Data collection and clinical evaluation

Demographic information was recorded for all eligible patients, and clinical details were obtained from patient records. Data included age, sex, duration and onset of symptoms, presence and pattern of diplopia, associated pain, history of trauma, systemic illnesses such as diabetes mellitus and hypertension, and relevant neurological or visual history.

All patients had undergone a comprehensive general and systemic examination to identify vascular risk factors and possible indicators of neurological disease. A complete ophthalmic evaluation had been performed by an ophthalmologist, including assessment of best-corrected visual acuity, color vision, ocular alignment in primary position and in cardinal gazes, and pupillary reactions to exclude involvement of other cranial nerves.

Anterior segment examination was performed using slit-lamp biomicroscopy, and posterior segment evaluation was performed using direct and indirect ophthalmoscopy.

Ocular motility assessment and ancillary tests

Prism bar cover testing had been performed to quantify ocular deviation in the primary position and in all cardinal positions of gaze, and the degree and extent of limitation of ocular movements were documented.

Diplopia charting using red-green goggles and diplopia field testing were performed in cooperative patients who were able to reliably report diplopia. These assessments enabled objective documentation of the pattern, severity, and progression of ocular misalignment and diplopia.

Investigations and imaging

Neuroimaging using CT or MRI of the brain and orbit was performed based on clinical indications. Neuroimaging was recommended in patients younger than 50 years, in those presenting with atypical clinical features such as severe pain, additional neurological deficits, progressive worsening of ocular motility restriction, history of trauma, clinical suspicion of infection or inflammation, or absence of clinical improvement during follow-up. Imaging findings were used to identify structural, inflammatory, vascular, or compressive causes of LR palsy.

Laboratory investigations were conducted when clinically indicated and included fasting blood glucose levels, glycated hemoglobin (HbA1c), and blood pressure assessment, along with other targeted investigations when systemic infection, inflammatory disease, or other secondary etiologies were clinically suspected. Cases were classified as microvascular ischemia when patients had established vascular risk factors such as diabetes mellitus or hypertension, no evidence of structural lesions on neuroimaging when performed, and a clinical course consistent with spontaneous improvement during follow-up.

Management

Management strategies were determined according to the underlying etiology and severity of clinical presentation. Patients with presumed microvascular ischemic LR palsy were managed conservatively, with emphasis on control of systemic risk factors such as diabetes mellitus and hypertension in coordination with appropriate medical care. Symptomatic management of diplopia included temporary occlusion therapy or prism correction when required to improve visual comfort during the recovery period.

Patients with traumatic, infectious, inflammatory, or compressive etiologies were managed according to the underlying cause. Appropriate medical therapy, referral for neurological evaluation, or neurosurgical consultation was undertaken when indicated based on clinical findings and imaging results. Management decisions were individualized based on the etiology identified during clinical evaluation and investigation.

Follow-up and outcome assessment

Patients were followed for a period of six months, and clinical recovery was assessed during follow-up visits based on ocular motility, ocular alignment, and resolution of diplopia. Follow-up evaluations were performed at regular clinical visits during the study period, and recovery status was documented at the final follow-up assessment. The primary outcome of the study was recovery of LR muscle function, while secondary outcomes included degree of improvement in ocular motility, persistence or resolution of diplopia, and overall recovery status at six months.

Recovery was classified using predefined criteria. Complete recovery was defined as full restoration of ocular abduction with complete resolution of diplopia. Partial recovery was defined as improvement in ocular movements with residual limitation of abduction or persistent diplopia. No recovery was defined as persistence of ocular motility restriction and diplopia at the final follow-up visit.

Patients who did not return for follow-up were recorded as lost to follow-up and were excluded from the final outcome assessment.

Sample size calculation

The sample size was calculated based on the expected prevalence of a specific etiology of LR palsy, cavernous sinus thrombosis, estimated at 4.8% from previous literature. Using a 95% confidence level and an absolute precision of 8%, the required sample size was calculated as 29 patients. The following formula was used:

$n=\frac{Z^2 p q}{d^2}$

Where, $n$ denotes the required sample size, $Z$ represents the standard normal deviate corresponding to the chosen level of significance $(\alpha)$, $p$ is the estimated population proportion, $q=1-p$, and $d$ is the absolute margin of error (precision).

Statistical analysis

All collected data were entered into Microsoft Excel (Microsoft Corp., Redmond, WA, USA) for organization and preliminary processing. Statistical analysis was performed using SPSS version 27 (IBM Corp., Armonk, NY, USA). Continuous variables were summarised using mean, standard deviation, median, and interquartile range, while categorical variables were presented as frequencies and percentages. The analysis was primarily descriptive, as the objective of the study was to characterise the clinical profile, etiological factors, and recovery outcomes of isolated LR palsy. Results were presented using tables and charts to enhance clarity and interpretability.

## Results

A total of 29 patients diagnosed with isolated LR palsy were included in the study and analyzed in detail. The results are presented under demographic characteristics, clinical presentation, ocular findings, imaging results, etiological distribution, and recovery outcomes.

Demographic characteristics and clinical presentation

The study population consisted of 29 adult patients. The mean age of the patients was 48.17 years, with an age range spanning from 19 to 80 years. The majority of patients were middle-aged adults, with the highest number falling within the 41-50-year age group. Gender distribution showed a near-equal representation, with 15 males accounting for 48.3% of the cohort and 14 females comprising 51.7%. Laterality analysis revealed that the left eye was more commonly affected than the right eye. Overall, 19 (65.5%) patients had involvement of the left eye, whereas 10 (34.5%) patients presented with right eye involvement.

The most common presenting complaint among patients was horizontal diplopia, which was reported by approximately 90% of the cases. Diplopia was typically worse on gaze toward the affected side, consistent with LR muscle dysfunction. Other presenting symptoms were minimal, as all cases represented isolated LR palsy without involvement of other cranial nerves.

Age distribution

Overall, two (6.9%) patients were aged 20 years or younger. Four (13.8%) patients each were noted in the 21-30-year and 31-40-year age groups. The highest frequency was observed in the 41-50-year age group, which included six (20.7%) patients. Five (17.2%) patients each were present in the 51-60-year and 61-70-year age groups. Three (10.3%) patients were aged above 70 years. This distribution demonstrates a predominance of isolated LR palsy in middle-aged and older adults. Age distribution of the patients is summarized in Table 1.

**Table 1:** Age distribution of the patients.

Age group (years)	Frequency	Percentage (%)
≤20	2	6.9
21–30	4	13.8
31–40	4	13.8
41–50	6	20.7
51–60	5	17.2
61–70	5	17.2
≥ 71	3	10.3
Total	29	100.0

**Figure 1:**
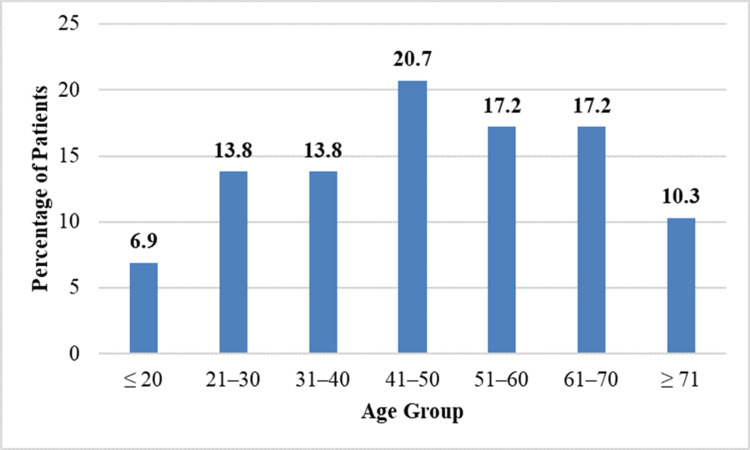
Age distribution of patients with isolated lateral rectus palsy.

Figure 1 shows the age distribution of patients with isolated LR palsy.

Gender distribution

Among the 29 patients, 14 (48.3%) were female, and 15 (51.7%) were male, indicating a slight male predominance, though the difference was minimal and not clinically significant. Table 2 represents the gender distribution of the study population.

**Table 2:** Gender distribution of the patients.

Gender	Frequency	Percentage (%)
Female	14	48.3
Male	15	51.7
Total	29	100.0

**Figure 2:**
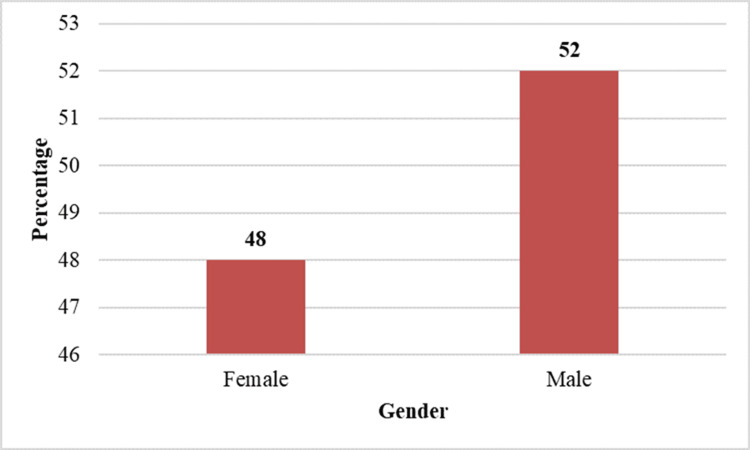
Gender distribution of patients with isolated lateral rectus palsy.

Figure 2 shows the gender distribution of the study population.

Laterality of eye involvement

Right eye involvement was observed in 10 (34.5%) patients, while left eye involvement was present in 19 (65.5%) patients. No cases of bilateral LR palsy were identified, as bilateral involvement was excluded by study criteria. Table 3 represents the laterality of eye involvement in isolated LR palsy.

**Table 3:** Laterality of eye involvement.

Eye involved	Frequency	Percentage (%)
Right	10	34.5
Left	19	65.5
Total	29	100.0

**Figure 3:**
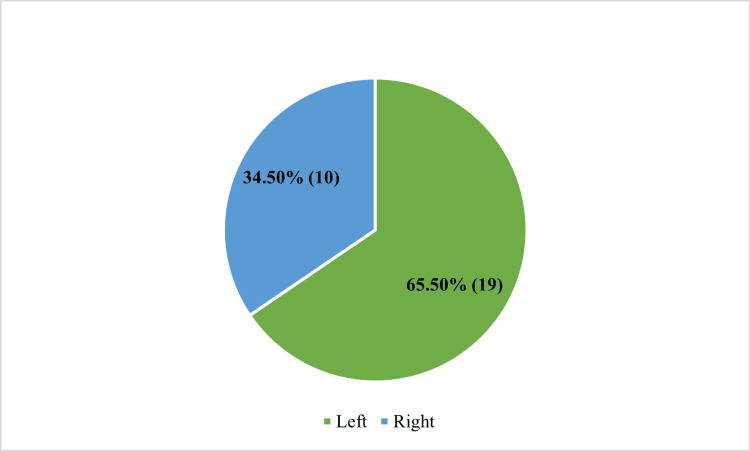
Laterality of eye involvement in isolated lateral rectus palsy.

Figure 3 shows the laterality of eye involvement in isolated LR palsy.

Visual acuity and fundus findings

Visual acuity assessment revealed that most patients had normal vision. Sixteen (48.3%) patients had best-corrected visual acuity of 6/6, while 11 (37.9%) patients had visual acuity of 6/9. Only one (10.3%) patient had reduced visual acuity of 6/18. Fundus examination in the majority of patients was normal. The single patient with reduced visual acuity demonstrated papilledema on fundoscopy, and this patient was subsequently diagnosed with cavernous sinus thrombosis as the underlying etiology. Visual acuity was preserved in most cases, reflecting the isolated nature of LR involvement without optic nerve pathology. Table 4 represents the visual acuity distribution among the patients.

**Table 4:** Visual acuity distribution of the patients.

Visual acuity	Frequency	Percentage (%)
6/6	16	48.3
6/9	11	37.9
6/18	1	10.3
Total	29	100.0

Primary deviation and ocular motility

Overall, 16 (55.2%) patients had a primary deviation of 20 prism diopters (PD). Ten (34.5%) patients demonstrated a deviation of 25 PD. Two (6.8%) patients had a deviation of 30 PD, while one (3.4%) patient showed a larger deviation of 45 PD. These findings indicate that most patients presented with mild to moderate esodeviation in the primary position. The range and restriction of ocular movements were documented in all cases and confirmed limitation of abduction in the affected eye. Table 5 represents the primary deviation measured in the affected eye.

**Table 5:** Primary deviation in the affected eye. PD: prism diopters

Primary deviation	Frequency	Percentage (%)
20 PD	16	55.2
25 PD	10	34.5
30 PD	2	6.8
45 PD	1	3.4
Total	29	100.0

**Figure 4:**
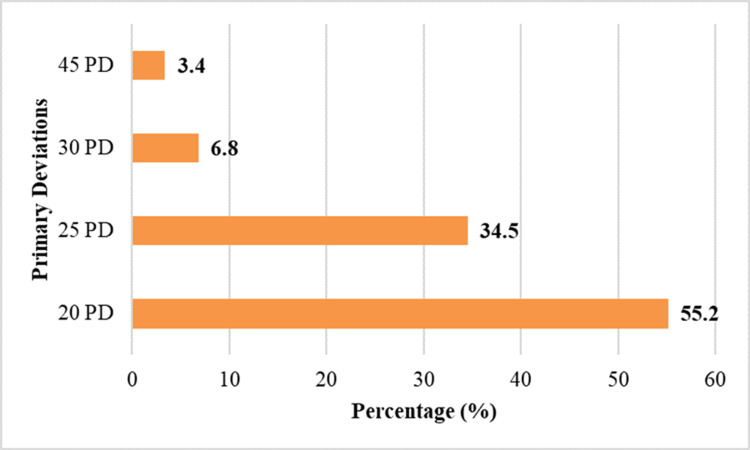
Distribution of primary deviation in the affected eye. PD: prism diopters

Figure 4 shows the distribution of primary deviation in the affected eye.

Pupillary status and diplopia testing

Pupillary reactions were normal in 28 (96.6%) patients. One (3.4%) patient exhibited papilledema, correlating with raised intracranial pressure due to cavernous sinus thrombosis. Field defect testing using diplopia charting and field tests was performed in cooperative patients. All patients demonstrated negative field defect testing, confirming the absence of visual field involvement. Table 6 represents the distribution of pupillary status among patients with isolated LR palsy.

**Table 6:** Pupillary status of the patients.

Pupillary status	Frequency	Percentage (%)
Normal	28	96.6
Papilledema	1	3.4
Total	29	100.0

Table 7 represents the field defect testing (FDT) results in patients with isolated LR palsy.

**Table 7:** Field defect testing (FDT) results.

FDT result	Frequency	Percentage (%)
Negative	29	100.0
Total	29	100.0

Neuroimaging findings

Neuroimaging was normal in most patients, while abnormal findings in the remaining cases reflected a wide etiological spectrum. The abnormalities broadly represented traumatic lesions, infectious or inflammatory processes, vascular pathology involving the cavernous sinus, intracranial hemorrhage, and a neoplastic lesion. These findings indicate that isolated lateral rectus palsy may be associated with diverse underlying intracranial and orbital pathologies, and the detailed CT and MRI results are summarized in Table 8.

**Table 8:** CT/MRI findings in patients with isolated LR palsy. CSOM: chronic suppurative otitis media; LR: lateral rectus

CT/MRI finding	Frequency	Percentage (%)
Normal	18	62.0
Basal skull bone fracture of the clivus and the temporal petrous ridge	1	3.4
Cavernous sinus thrombosis	2	6.9
Craniopharyngioma	1	3.4
Localised abscess along LR muscle with orbital cellulitis	1	3.4
Midbrain tuberculoma	1	3.4
Orbital cellulitis with acute dacryoadenitis	1	3.4
Petrositis with CSOM	1	3.4
Subarachnoid hemorrhage	2	6.8
Temporal bone fracture with hemotympanum	1	3.4
Total	29	100.0

Etiological distribution

Microvascular ischemia constituted the predominant etiological category in this cohort, accounting for 15 of the 29 cases (51.7%), whereas the remaining patients were distributed among infectious or inflammatory causes (four cases, 13.8%), traumatic etiologies (four cases, 13.8%), cavernous sinus thrombosis (three cases, 10.3%), idiopathic causes (two cases, 6.9%), and neoplastic pathology (one case, 3.4%). Microvascular ischemia included cases associated with diabetes mellitus, hypertension, or both systemic conditions. Overall, vascular mechanisms formed the largest subgroup, followed by inflammatory and traumatic causes, with smaller contributions from compressive and idiopathic etiologies. The detailed distribution of individual diagnoses is presented in Table 9.

**Table 9:** Etiological distribution of isolated lateral rectus palsy among study participants (n = 29). Microvascular ischemia was diagnosed in patients with vascular risk factors and absence of structural lesions on imaging when performed. DM: diabetes mellitus; HTN: hypertension; LR: lateral rectus

Etiology	Frequency	Percentage (%)
Idiopathic	2	6.9
Infectious/Inflammatory causes	4	13.8
Microvascular ischemia – DM	7	24.1
Microvascular ischemia – HTN	7	24.1
Microvascular ischemia – DM + HTN	1	3.4
Cavernous sinus thrombosis	3	10.3
Trauma (road traffic accident)	4	13.8
Tumor (compressive lesion)	1	3.4
Total	29	100.0

Recovery at three months

Recovery status at three months follow-up is summarized in Table 10. At three months, 21 (72.4%) patients had achieved full recovery. Partial recovery was observed in five (17.2%) patients. Three (10.3%) patients continued to have a persistent deficit at the three-month follow-up. These findings suggest that a majority of patients showed significant improvement within the first three months. Table 10 represents the recovery status of patients at the three-month follow-up.

**Table 10:** Recovery status at three-month follow-up. Complete recovery was defined as full restoration of ocular abduction with complete resolution of diplopia. Partial recovery indicated improvement in ocular movements with residual limitation or persistent diplopia. No recovery referred to persistence of ocular motility restriction and diplopia at the final follow-up visit.

Recovery status	Frequency	Percentage (%)
Complete recovery	21	72.4
Partial recovery	5	17.2
No recovery (persistent deficit)	3	10.3
Total	29	100.0

Recovery at six months

Recovery outcomes at six months are detailed in Table 11. At the end of six months, 19 (82.8%) patients had achieved full recovery. Partial recovery was observed in two (6.9%) patients, while one (3.4%) patient had a persistent deficit. Seven (24.1%) patients were lost to follow-up at six months. Among patients who completed follow-up, the majority demonstrated complete resolution of symptoms and restoration of ocular motility. The patients with persistent deficits were primarily those with traumatic or compressive etiologies. Table 11 represents the recovery status of patients at six-month follow-up.

**Table 11:** Recovery status at the six-month follow-up. Complete recovery was defined as full restoration of ocular abduction with complete resolution of diplopia. Partial recovery indicated improvement in ocular movements with residual limitation or persistent diplopia. No recovery referred to persistence of ocular motility restriction and diplopia at the final follow-up visit. Patients who did not return for follow-up were categorized as lost to follow-up and were excluded from final outcome assessment.

Recovery status	Frequency	Percentage (%)
Complete recovery	19	65.5
Partial recovery	2	6.9
No recovery (persistent deficit)	1	3.4
Lost to follow-up	7	24.1
Total	29	100.0

**Figure 5:**
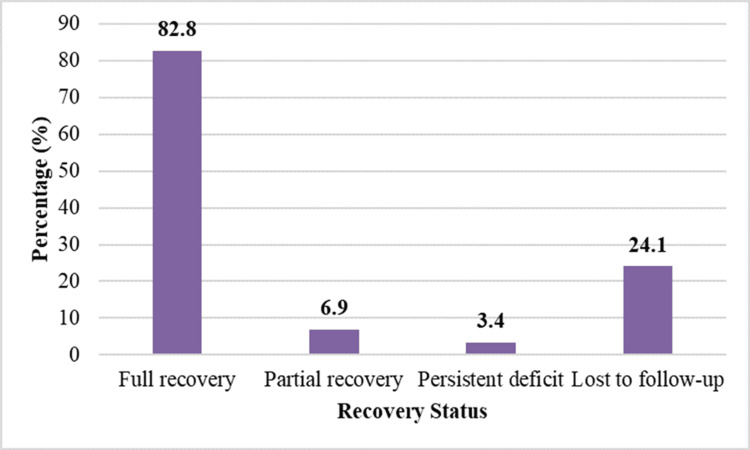
Recovery status of patients at the six-month follow-up.

Figure 5 shows the recovery status of patients at six-month follow-up.

Imaging illustrations

**Figure 6:**
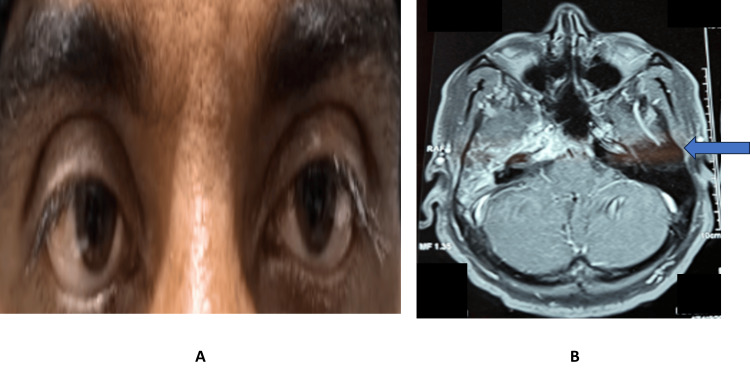
Clinical and imaging features of isolated lateral rectus palsy. (A) Right eye lateral rectus palsy. (B) CT scan showing petrositis image.

The results demonstrate that isolated LR palsy in adults predominantly affects middle-aged individuals, commonly presents with horizontal diplopia, and is most frequently caused by microvascular ischemia. The majority of patients show favorable recovery within six months, particularly those with vascular or inflammatory etiologies, while traumatic and compressive causes are associated with poorer outcomes. At six months, 19 patients had full recovery, two patients had partial recovery, and one patient had a persistent deficit. We had lost seven patients to follow-up at six months. Representative clinical images are included to demonstrate typical presentations and imaging findings. Figure 6 illustrates right eye LR palsy with a corresponding CT scan showing petrositis.

**Figure 7:**
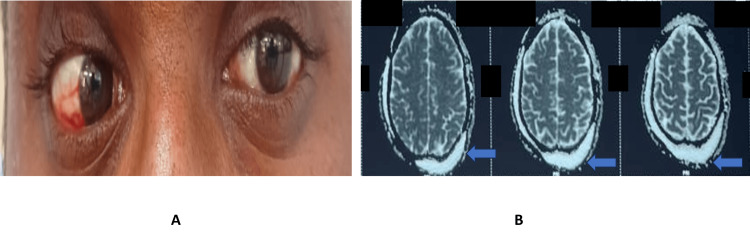
Clinical photograph and MRI of the brain demonstrating isolated LR palsy. (A) Right eye lateral rectus palsy. (B) MRI showing subarachnoid hemorrhages in sulci of frontoparietal lobes.

Figure 7 depicts right eye LR palsy with MRI demonstrating subarachnoid hemorrhages in the sulci of the frontoparietal lobes.

**Figure 8:**
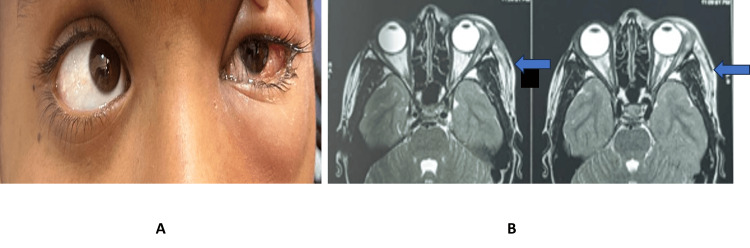
Clinical photograph and MRI findings in isolated LR palsy. (A) Left eye lateral rectus palsy. (B) MRI showing localized orbital abscess with orbital cellulitis with acute dacryoadenitis.

Figure 8 shows left eye LR palsy with MRI findings of a localized orbital abscess associated with orbital cellulitis and acute dacryoadenitis.

## Discussion

This study has examined the clinical characteristics of isolated LR palsy in 29 adults. The age structure was reasonably balanced between the genders and slightly biased toward men. The largest number of patients were in the middle-aged category, especially those who were between 41 and 50 years, followed by those who were 51-70 years old. The median age at the onset was 48.17 years, which showed that the isolated LR palsy is more prevalent in middle-aged and older patients. The involvement of the left eye was higher than that of the right eye, which is also reported in other clinical series.

The most frequent etiology in the study was microvascular disease, and it was found in little more than half of the cases [11]. The largest proportion of this population was made up of patients with diabetes mellitus and hypertension, confirming the fact that vascular risk factors are associated with sixth nerve palsy [12]. The most common vascular cause was diabetic mononeuropathy, closely followed by hypertension, highlighting the role played by systemic metabolic conditions in cranial nerve ischemia [13]. This close correlation justifies the need to conduct regular systemic assessment and metabolic screening in adults with isolated LR palsy [14]. There are also similar results on large clinical and population-based studies to assess ocular motor nerve palsies [15].

This cohort also had excellent representation of non-vascular causes. A significant percentage of cases was explained by traumatic etiology, which is mostly associated with road traffic accidents [16]. Various causes of infections and inflammation were detected in many patients, such as orbital cellulitis, acute dacryoadenitis, petrositis, and midbrain tuberculoma [17]. Another significant etiology was cavernous sinus thrombosis, and it is important to carefully evaluate the patient in cases of a high level of pain, systemic manifestations, or abnormal clinical features [18]. A few of the patients were neoplastic, with craniopharyngioma, and a minority were idiopathic with extensive investigations [19].

These results highlight the observation that microvascular ischemia is the most common cause of isolated LR palsy, yet clinicians should be very keen to note other possible etiologies [20]. Less common clinical causes are traumatic, inflammatory, infectious, and compressive, which may need urgent research and specific treatment [3]. Younger patients or those who develop symptom progression, pain, or other neurological manifestations are more likely to experience such etiologies, which require an immediate neuroimaging test and expert care referral [9].

The observation in this research was in line with the typical characteristics of isolated LR palsy. The most frequent presenting complaint was that of horizontal diplopia that was exacerbated by gaze on the side ipsilateral to the affected side [6]. The primary position result esotropia and abduction restriction were universal results and indicated failure of the abducens nerve on its intracranial or orbital pathway [1]. In the cases that were really isolated, there was no further cranial nerve involvement or long-tract neurological features, which aids in distinguishing these patients in comparison to those with a more widespread neurological disease [8].

In the majority of the patients, visual acuity was spared, indicating the isolated effect of the LR muscle without ocular nerve pathology [5]. Reduced visual acuity linked with papilledema caused by elevated intracranial pressure was detected in only one patient, which underlines the utility of fundus examination in diagnosing secondary causes [10]. Almost all patients had normal pupillary responses, which again indicates that it was a case of isolated sixth nerve palsy and not touching on the adjacent cranial nerves [2].

The results of recovery were positive in this study, as a rule. At the six-month follow-up, two-thirds of the patients had recovered fully, with a smaller proportion having partial recovery or never recovered [14]. Etiologies with the largest recovery rates were microvascular, infectious, and inflammatory, which were in line with the generally benign natural history of the etiologies [11]. Traumatic and compressive causes, in contrast, were linked to poorer recovery, especially in cases where there was a major head trauma or a structural lesion [16]. The extent of primary deviation at presentation appeared to be associated with recovery patterns in this cohort, with lower deviations generally observed among patients who demonstrated better outcomes [12].

The general recovery process noted is in line with previous research that reported isolated LR palsy, which is commonly of a microvascular nature, follows a self-limiting course with spontaneous improvement in a few months [15]. It also did not seem that age or sex had a significant effect on the outcome of recovery, and no distinct difference was noted between total and partial palsy regarding prognosis [7]. The results also confirm conservative management and close follow-up in select patients [4].

The underlying etiology of isolated LR palsy is an important factor influencing the observed recovery patterns. In this study and previous reports, microvascular palsies generally demonstrated favorable recovery patterns, whereas palsies associated with neoplastic, traumatic, or persistent inflammatory causes tended to show more variable or less favorable outcomes [18]. Depending on the pathology, such cases can require certain interventions, such as surgical, medical, or antimicrobial therapy [20].

The study had a single-center design with a limited sample size, which could be a limitation to generalizability, and population studies usually depend on comorbidity coding as opposed to standardised diagnostic assessment. Clinical implications consist of screening for diabetes and other vasculopathic risk factors in all adults with isolated LR palsy and the maximization of glycemic and cardiovascular control. Outpatient follow-up and symptomatic treatment could be sufficient in the short term in patients who have typical characteristics of microvascular palsy. Assessment of other causes, including trauma, infection, cavernous sinus pathology, or neoplasm, should be based on clinical history, physical examination, and imaging evidence, and treatment given to the underlying etiology in case of identification. Because this investigation used a descriptive observational design without inferential statistical testing, the findings should be interpreted as patterns observed within this cohort rather than evidence of causal or predictive relationships.

Strengths and limitations of the study

The present study has several methodological strengths. Patients were included consecutively during the study period, which helped reduce selection bias and ensured representation of cases encountered in routine clinical practice. In addition, a structured clinical evaluation with standardized ophthalmic examination and clearly defined recovery categories allowed consistent documentation of clinical findings and outcomes. The use of systematic follow-up assessments and table-based presentation of demographic characteristics, etiologies, and recovery outcomes further improves the clarity and transparency of the data.

However, a few limitations must be considered when interpreting the results. The relatively small sample size limits the statistical power of the study and restricts the ability to perform subgroup analyses or identify independent predictors of recovery. The study was also conducted at a single tertiary care center, which may limit the generalizability of the findings to other populations or healthcare settings. In addition, the statistical analysis was primarily descriptive and did not include inferential or multivariable analysis, which limits the ability to explore causal relationships between etiological factors and recovery outcomes.

Another important limitation is the loss to follow-up observed in a proportion of patients during the six-month follow-up period, which may introduce potential follow-up bias. Patients lost to follow-up may have experienced recovery or persistent deficits, and their absence from the final assessment could influence the estimated recovery rates. Despite these limitations, the study provides useful descriptive insights into the clinical profile, etiological distribution, and recovery outcomes of isolated LR palsy in adults presenting to a tertiary care center.

## Conclusions

This study describes the clinical profile and etiological distribution of isolated LR palsy in adults presenting to a tertiary care center. Microvascular ischemia associated with diabetes mellitus and hypertension was the most frequently observed etiology in this cohort. Many of these cases demonstrated a non-progressive course and showed improvement during follow-up, supporting the role of conservative management and close clinical monitoring in appropriately selected patients. At the same time, the findings emphasize the importance of recognizing atypical clinical patterns, because presentations characterized by progression of symptoms, severe pain, bilateral involvement, associated neurological deficits, or lack of clinical improvement may indicate non-ischemic etiologies such as compressive, inflammatory, infectious, or neoplastic causes. In such situations, timely neuroimaging and specialist referral are important for accurate diagnosis and appropriate management. Given the descriptive observational design, small sample size, and single-center setting, these findings should be interpreted with caution and cannot be generalized to all populations. Further studies with larger cohorts and analytical statistical methods are required to better characterise etiological patterns and recovery outcomes in isolated LR palsy.
